# Unsolved Issues in the Integrated Histo-Molecular Classification of Endometrial Carcinoma and Therapeutic Implications

**DOI:** 10.3390/cancers16132458

**Published:** 2024-07-04

**Authors:** Elisabetta Kuhn, Donatella Gambini, Letterio Runza, Stefano Ferrero, Giovanna Scarfone, Gaetano Bulfamante, Ayse Ayhan

**Affiliations:** 1 Department of Biomedical, Surgical and Dental Sciences, University of Milan, 20122 Milan, Italy; stefano.ferrero@unimi.it (S.F.); gaetano.bulfamante@unimi.it (G.B.); 2 Pathology Unit, Foundation IRCCS Ca’ Granda Ospedale Maggiore Policlinico, 20122 Milan, Italy; letterio.runza@policlinico.mi.it; 3 Department of Neurorehabilitation Sciences, Casa di Cura Igea, 20144 Milan, Italy; d.gambini@casadicuraigea.it; 4 Gynecology Oncology Unit, Foundation IRCCS Ca’ Granda Ospedale Maggiore Policlinico, 20122 Milan, Italy; giovanna.scarfone@policlinico.mi.it; 5 Human Pathology and Molecular Pathology, TOMA Advanced Biomedical Assays S.p.A., 21052 Busto Arsizio, Italy; 6 Department of Tumor Pathology, Hamamatsu University School of Medicine, Hamamatsu 431-3192, Japan; ayseayhanjp@gmail.com; 7 Department of Gynecology and Obstetrics, Johns Hopkins University School of Medicine, Baltimore, MD 21218, USA

**Keywords:** endometrial cancer, endometrial, classification, history, targeted therapy, checkpoint inhibitors

## Abstract

**Simple Summary:**

In the past few years, the endometrial cancer field has been revolutionized, following the integration of molecular classification into clinical practice. Endometrial carcinoma is currently molecularly categorized into four main subgroups: POLE-mutated, mismatch repair-deficient, p53-mutated, and no specific molecular profile. The POLE-mutated subgroup is characterized by an excellent prognosis notwithstanding bad conventional prognostic factors, including high-grade and *TP53* mutations. On the other hand, the p53-mutated subgroup demonstrates the worst outcome. However, the remaining subgroups are more prevalent and display variable clinical outcomes. In this review, we describe the evolution of the endometrial carcinoma classification, its strengths and limitations, clinical implications, and foreseen perspectives.

**Abstract:**

Endometrial carcinoma (EC) is the most frequent gynecological cancer, with an increasing incidence and mortality in recent times. The last decade has represented a true revolution with the development of the integrated histo-molecular classification of EC, which allows for the stratification of patients with morphologically indistinguishable disease into groups with different prognoses. Particularly, the POLE-mutated subgroup exhibits outstanding survival. Nevertheless, the indiscriminate application of molecular classification appears premature. Its prognostic significance has been proven mainly in endometrioid EC, the most common histotype, but it has yet to be convincingly confirmed in the other minor histotypes, which indeed account for a relevant proportion of EC mortality. Moreover, its daily use both requires a mindful pathologist who is able to correctly evaluate and unambiguously report immunohistochemical staining used as a surrogated diagnostic tool and is hampered by the unavailability of *POLE* mutation analysis. Further molecular characterization of ECs is needed to allow for the identification of better-tailored therapies in different settings, as well as the safe avoidance of surgery for fertility preservation. Hopefully, the numerous ongoing clinical trials in the adjuvant and metastatic settings of EC will likely produce evidence to refine the histo-molecular classification and therapeutic guidelines. Our review aims to retrace the origin and evolution of the molecular classification for EC, reveal its strengths and limitations, show clinical relevance, and uncover the desired future developments.

## 1. Introduction

Endometrial carcinoma (EC) is the most frequent gynecologic malignancy in developed countries but only the seventeenth most frequent cause of cancer-related death [1]. Importantly, after an astonishing decline over the twentieth century, the incidence and mortality of endometrial cancer have progressively increased in the last decade [1,2]. Moreover, the short-term outcome is ominous, and current therapeutic approaches are ineffective in patients with advanced or recurring EC.

The initial rudimentary site-based classification of tumors has progressively evolved, with the crucial contribution of pathologists and epidemiologic studies, disclosing the multitude of different tumor diseases that can affect each site, including the corpus uteri [3]. Nevertheless, Bokhman’s dualistic pathogenetic model has served as the primary conceptual framework for clinical and experimental research for at least three decades, simplifying the diverse histological appearance of ECs [4,5].

The advent and diffusion of automated and massive molecular techniques have revolutionized all fields of knowledge, particularly the biological sciences. In oncology, molecular characterization has allowed a better understanding of the myriads of tumor types, moving from a histopathological to a combined histo-molecular classification. For instance, the molecular genetic characterization has further displayed the variety of EC, recognizing at least four different intrinsic molecular types of EC with different prognostic and therapeutic impacts [6]. This new perspective is very appealing because it reduces the subjectivity of the diagnosis, but it is still immature and risky. In particular, the molecular classification was based on a multi-omic study of 343 ECs, including endometrioid, serous, and mixed histotypes, by the Cancer Genome Atlas (TCGA). Importantly, many histotypes were not included, and although an association between molecular types and EC histotypes has been reported, its prognostic impact in non-endometrioid EC is imperfect and still incompletely investigated.

The validity of a particular classification is confirmed at least decades after its proposal and use; it usually necessitates application and investigation until its long-lasting validation. The work of the last decade in EC started to intercept the flaws of molecular classification and dissect comparisons with earlier, long-lasting classifications. The novel, evolving concepts may be complicated for practicing clinicians, in particular pathologists and gynecologic oncologists. This review attempts to summarize the historical process that has led to the new integrated histo-molecular classification of EC, simplify the emerging concepts at the base of the new risk group stratification guiding therapeutic choices, and describe ongoing clinical trials.

## 2. Bokhman’s Pathogenetic Model

The first and traditional classification was anatomical, and it mainly distinguished between cervical and endometrial cancers. In 1983, Bokhman proposed the two-way pathogenetic model of endometrial cancer [4], derived from an elegant, prospective study on 366 patients with EC over a 20-year period. Bokhman’s model included pathogenetic type I ECs arising in obese women with hyperlipidemia and hyperestrogenism. These tumors, accounting for 65% of the study group, were well or moderately differentiated, associated with endometrial hyperplasia, superficial, highly sensitive to progestins, and had a good prognosis. Conversely, type II endometrial cancer, which accounted for the remaining 35% of cases, arose in non-obese women without metabolic and endocrine disorders, and showed poor differentiation, association with atrophic endometrium, deep myometrial invasion, frequent metastatic disease, progesterone resistance, and a poor prognosis.

From a histopathological standpoint, type I tumors broadly correspond to endometrioid ECs, while type 2 tumors are non-endometrioid ECs. This brilliant dualistic model has demonstrated its broad validity and general correctness and has represented an important conceptual framework lasting for at least three decades. It provided the basis for epidemiologic, statistical, clinical, and biological studies favoring education and scientific improvement in the EC field, and it is still applicable and used. Moreover, it was also further recovered in 2004 by Kurman and Shih and applied to ovarian cancer, with several opportune modifications [7,8,9]. It should be stressed that both are simplified models to outline relevant etiopathological and clinicopathological features with documented molecular correlations, rather than diagnostic terminology to use in daily practice.

Over time, the complex variety of endometrial malignancies in terms of clinical, histopathological, and molecular features is being refined and enriched with new entities with prognostic impact. In this new scenario, Bokhman’s dichotomic simplification demonstrates several limits, not being able to cover the full range of possibilities in only two categories. Apparently, both type I and type II tumors are not watertight compartments or homogenous groups but include entities with clinical, epidemiologic, histologic, and even molecular characteristics of both types. For example, high-grade endometrioid EC, even if it has typical clinical and epidemiological features of type I tumors, may demonstrate an ominous prognosis and even molecular features of type II tumors, such as *TP53* mutation (in about 30% of cases) or *HER2* amplification (in 0.2–25%) [10,11]. On the other hand, even the prototypical type II serous EC may follow atypical endometrioid hyperplasia in premenopausal women with hyperestrogenism. The same is true for most other high-grade hystotypes, including dedifferentiated, undifferentiated, clear cell carcinoma, and carcinosarcoma [12]. Therefore, this model should be reconsidered in light of recent knowledge and the updated WHO classification, with the hope of reaching an optimal and flawless simplification.

## 3. The WHO Classification

### 3.1. Historical Perspectives

The World Health Organization (WHO) classification represents a successful project aimed at uniformizing tumor classification worldwide, therefore serving as a standard reference for practical taxonomy among pathologists, clinicians, and scientists. The first attempt to standardize the histological classification of EC dates back to 1975, when the WHO published the first edition of “Histological typing of female genital tract tumours” [13]. This classification was international, site-specific, and primarily based on histopathological features, that is, the identifiable cell type and the architectural pattern, observable under a light microscope. Notably, other techniques available at that time, such as electronic microscopy, histochemistry, and immunohistochemistry (IHC), could both support the definition and help the diagnosis, but were not needed. In particular, IHC was becoming an extremely powerful tool for diagnostic pathologists, valuable to this day. The main purpose of this classification was to develop histological definitions of tumor types and a shared, uniform nomenclature to be progressively updated to integrate emerging knowledge due to new observations with standard or innovative techniques. The first classification was rudimentary but essential, distinguishing between five subtypes of epithelial malignant tumors of the corpus uteri: adenocarcinoma, clear cell adenocarcinoma, squamous cell carcinoma, adenosquamous adenocarcinoma, and undifferentiated carcinoma (Table 1) [13].

In 1994, almost twenty years later, the second edition was published, entrusted to the Classification and Nomenclature Committee of the International Society of Gynecological Pathologists with Dr. Scully as Chairman and involving 30 pathologists around the world [3]. The updated classification included the main histological types of EC that we still know and diagnose today (Table 1). The classification maintained a morphological framework and reflected the diagnostic progress due to the widespread use of IHC in pathologic diagnosis, considering the clinical and epidemiological relevance of tumor types.

The WHO third edition was supplemented with cytogenetics and molecular genetic profiling, which is the incorporation of genetic information that represents the initial step to a molecular-based classification for all organ tumors [14]. This new approach is clearly reflected in the title of the series, “Pathology and Genetics of Tumours”. Moreover, thanks to the multidisciplinary contribution of epidemiologists, radiologists, gynecologists, and oncologists, the volume on tumors of female genital organs, published in 2003, was enhanced by emerging epidemiologic, etiologic, prognostic, and IHC features. As a result, the format of this series appears to be significantly enriched, adding to formal classification schemes and definitions with ICD-O (international classification of diseases for oncology codes) comprehensive epidemiological, pathogenetic, clinical, imaging, pathological, prognostic, and predictive features, as well as differential diagnosis. Regarding EC, a villoglandular variant of endometrioid adenocarcinoma was added to the existing ones, and two new entities, transitional cell and small cell carcinomas, were recognized.

The following updated 2014 classification was conceptually in line with the previous one [15]. Semantically, the term “endometrioid carcinoma” was preferred to “endometrioid adenocarcinoma”, as for other entities, even if they remain interchangeable. Importantly, the 2014 WHO classification has inserted serous endometrial intraepithelial carcinoma (SEIC), dedifferentiated carcinoma, and neuroendocrine tumors among ECs. In particular, SEIC was introduced for its metastatic potential, despite its non-invasive nature. Two histotypes of EC were removed, namely transitional cell and squamous cell carcinomas. Moreover, minimal definitional changes have been made. Specifically, mixed cell adenocarcinoma must show several recognizable histologic types, accounting for at least 5% of the neoplasia, and must include a type II tumor, according to the Bokhman model. Finally, by definition, mucinous carcinoma must contain intracytoplasmic mucin in more than 50% of the neoplasia. 

### 3.2. Current WHO Classification

The most recent revision of the WHO classification of female genital tract tumors, published in 2020, emphasized key molecular features that allowed for both the refinement of conventional neoplasia categorization and the identification of new tumor entities [5]. Notably, the histopathological features maintain their primary role, but an integrated morphological–molecular approach is strongly recommended. In fact, the molecular classification, based on the Cancer Genome Atlas (TCGA) studies, complements the morphological classification of ECs due to its prognostic and predictive value (see below) and is incorporated in endometrioid EC [6]. In addition, the main changes include the introduction among ECs of mesonephric adenocarcinoma, mesonephric-like adenocarcinoma, mucinous carcinoma, intestinal type (successively revised to gastric type), and carcinosarcoma NOS, as well as the reintroduction of squamous cell carcinoma NOS as a separate entity and the removal of SEIC and neuroendocrine tumors [16]. Notably, the histological patterns of endometrioid EC, such as squamous differentiation, villoglandular, and secretory, are not reported in the classification for their irrelevant prognostic value. However, they are mentioned together with the other main patterns, including microglandular, spindle cell, sertoliform, and mucinous, as nuances of the morphological spectrum of endometrioid EC.

## 4. The Molecular Classification

Given the limitations of traditional histopathological subtyping and grading schemes with respect to reproducibility and prognostic capacity, particularly for high-grade ECs, ancillary techniques offer an opportunity for a more objective and reproducible tool for EC subclassification. Initially, IHC staining, and more recently, molecular markers, or a combination of both, have shown their utility in subclassifying ECs [17,18,19,20,21]. Understanding mutation patterns in different tumors may have diagnostic value for their subclassification, but more importantly, for both the risk stratification of patients and the identification of specific targets in order to guide the administration and development of targeted therapies.

**Figure 1:**
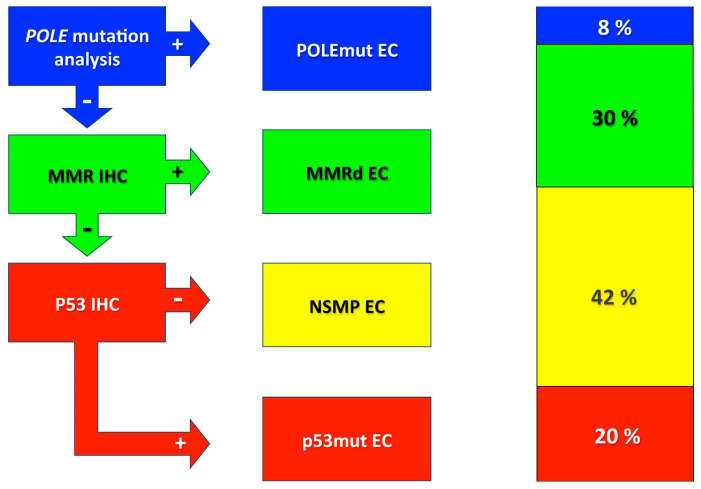
WHO diagnostic algorithm for molecular classification of ECs and, on the right, the relative approximative prevalence of different molecular subtypes. Acronyms: POLEmut, POLE-mutated; +, POLE pathogenically mutated or abnormal immunohistochemistry (IHC) pattern; -, POLE without pathogenic mutated or normal IHC pattern; IHC, immunohistochemistry; EC, endometrial carcinoma; MMR, mismatch repair proteins (MLH1, MSH2, MSH6, and PMS2); MMRd, mismatch repair-deficient; NSMP, no specific molecular profile.

In 2013, the TCGA published its seminal paper that, based on a multi-omic study, distinguished four main molecular subtypes of endometrial carcinomas: *POLE* (DNA Polymerase Epsilon) ultramutated, microsatellite instability hypermutated, copy-number low, and copy-number high [6]. Notably, these molecular differences underpin a biological and significant difference in progression-free survival (PFS). The main novelty was the identification of new mutations affecting the exonuclease domain of the *POLE* gene (which is a DNA repair gene), which were associated with an extremely favorable prognosis. On the other hand, the worst prognosis was associated with the copy-number high, whereas the remaining two subtypes, microsatellite instability hypermutated and copy-number low, demonstrated an intermediate prognosis. Subsequently, two research groups from Leiden and Vancouver, respectively, investigated a practical and simpler approach to molecularly classify EC, avoiding extensive, laborious, and out-of-reach technologies [22,23]. The Leiden group investigated a combination of IHC staining (including ARID1A, β-catenin, estrogen receptor, MLH1, MSH2, MSH6, p53, PMS2, progesterone receptor, and PTEN) and molecular analyses, including microsatellite instability (Promega) and targeted hotspot mutational analysis in 15 genes frequently mutated in EC, including *POLE* and *TP53.* This study focused on 116 high-risk invasive ECs (non-endometrioid EC of stages I–III, or endometrioid EC of any grade and stage II-III or grade 3 stage IA with documented lymphovascular space invasion or stage IB) and included 18 clear cell ECs, in addition to 86 endometrioid ECs and 12 serous ECs. The Leiden study identified four molecular subgroups: 38% of ECs had no specific molecular profile (NSMP), 34% were p53-mutant (p53mut), 16% were microsatellite instable (MSI), and 12% were POLE-mutant (POLEmut). Prognostically, both POLEmut and MSI groups did not have distant metastases and had significantly better 5-year PFS compared with both NSMP and p53mut and improved overall survival (OS) compared with p53mut. Clear cell ECs were a molecularly heterogeneous group composed mainly of p53mut (44%), followed by NSMP (39%), MSI (11%), and POLEmut (6%). On the other hand, the Vancouver study showed that a combination of five IHC stainings, routinely used in most pathological laboratories, associated with the mutational analysis for *POLE* provided a molecular classifier, called the ProMisE model (Proactive Molecular Risk Classifier for Endometrial Cancer), with prognostic value in OS [22]. This cheap and simple approach classified ECs as POLEmut, mismatch repair-deficient (MMRd), p53 abnormal (p53abn), and p53 wild-type (p53wt) subgroups. Other studies followed these original studies that confirmed the validity of simplified molecular classifier models and allowed to refine the molecular algorithm. Currently, the WHO recommends a stepwise molecular diagnostic algorithm: initially performing the *POLE* mutational analysis, followed by MMR IHC, and eventually p53 IHC (Figure 1).

This sequence of tests allows us to classify ECs in POLEmut, MMRd, p53mut, and NSMP. These categories are not identical but roughly correspond to the TCGA molecular subtypes, respectively, *POLE* ultramutated, MSI hypermutated, copy-number high, and copy-number low, and demonstrate prognostic significance. Specifically, POLEmut has an extremely favorable prognosis, p53mut has the worst outcome, and both the MMRd and NSMP demonstrate an intermediate prognosis. In addition, this classification predicts recurrence and possibly a response to therapy. 

### 4.1. Advantages

The molecular classification allows for diagnostic reproducibility and objectivity, reducing diagnostic inaccuracy and inconsistency. Specifically, the TCGA classification demonstrates strong concordance both in interlaboratory studies and between biopsic and surgical samples [24,25]. Conversely, histopathological diagnosis is affected by remarkable interobserver variability, particularly in high-grade histotypes, on which also expert gynecopathologists demonstrate a high rate of disagreement [19,26]. The TCGA molecular classification of ECs carries independent prognostic value as well as predictive power of therapy response [27]. However, the routine application of TCGA molecular methods is expensive and cumbersome for daily pathological practice. As a consequence, the hierarchical diagnostic approach based on *POLE* mutation analysis together with IHC surrogates has been embraced and recommended by the latest WHO classification. Therefore, the WHO algorithm method is pragmatic and time-effective; it could surrogate more complex, expensive molecular studies and could be applied to routine formalin-fixed, paraffin-embedded samples in daily practice by many standard pathology laboratories.

An additional advantage of the molecular classification derives from its power of segregation; that is, it segregates the tumors based on “mutation priority”, which is reflected in the stepwise WHO algorithm. This is specifically important in so-called multiple classifiers, harboring more than one molecular alteration among those defining the EC molecular subgroups [28]. POLEmut and MMRd tumors are characterized by inaccuracy in DNA repair, associated with a high number and variety of casual mutations falling into cell cycle checkpoints, chromatin regulatory genes, and proliferation, but also *TP53* and MMR genes. Based on survival analyses, the POLEmut patient group has a better prognosis, regardless of whether they also harbor MMR or *TP53* mutations [28]. Similarly, the MMR deficiency dictates the prognosis, even in the presence of concurrent *TP53* mutations. On the other hand, in patients classified as p53mut, the *TP53* mutation is the main tumor driver and should not harbor either MMR or *POLE* mutations as the initiating factor.

The molecular classification is able to stratify high-grade endometrioid EC into groups with very different outcomes. Being integrated into the risk stratification of ECs due to its prognostic ability (see below), in otherwise morphologically undistinguishable diseases, the molecular classification allows for avoiding both unnecessary overtreatment, for example, in POLEmut patients, and dangerous undertreatment in molecularly aggressive p53mut ECs.

### 4.2. Limitations

#### 4.2.1. Immunohistochemical Staining as a Surrogate

EC molecular classification, as recommended by the WHO, is routinely performed using IHC staining as a surrogate of genomic techniques. Both p53 and MMR immunostainings, the latter including MLH1, MSH2, MSH6, and PMS2, are available in most pathology laboratories and are strongly, but not perfectly, concordant with more complex molecular analyses detecting DNA mutations, promoter methylation of the *MLH1* gene, and MSI. Agreement between *TP53* mutation analysis and p53 IHC is attested at over 90% in most studies [29,30]. Similarly, concordance between the MSI assay or next-generation sequencing and MMR IHC usually exceeds 93% [31,32,33]. Regardless, the validity and reproducibility of IHC depend on adequate technique optimization, quality control, and correct interpretation by conscious pathologists. 

IHC identifies different patterns of p53 protein distribution that correlate with *TP53* gene mutation status. Wild-type *TP53* determines a sparse and variably intense positivity in a minority of cells (wild-type pattern), while mutated *TP53* induces five different IHC patterns: (1) overexpression characterized by intense and diffuse nuclear positivity in at least 80% of neoplastic cells (due to missense mutation); (2) null pattern, showing complete negativity (due to truncating mutations); (3) cytoplasmic pattern, characterized by variable nuclear positivity associated with obvious cytoplasmic positivity (due to mutations affecting the p53 nuclear translocator domain); (4) wild-type pattern (due to some splice site mutations); (5) subclonal pattern, consisting of a combination of the wild-type pattern with any mutation pattern as a consequence of an emerging mutation in a subpopulation of EC cells [34]. In unselected ECs, the proportion of different p53-mutated patterns is still to be determined, but, similar to tubo-ovarian high-grade serous carcinoma, the large majority of *TP53* mutations identified by TCGA (71%) in ECs are missense, usually linked to diffuse and strong overexpression. In a high-risk EC cohort, the mutated pattern was overexpression in 68%, subclonal in 21%, null in 9%, and cytoplasmic in 2% of evaluated cases [30]. 

Interobserver disagreement concerning p53 evaluation may affect routine pathology practice and eventually clinical decisions. Misinterpretation issues may derive from delayed fixation that may cause reduced expression, cytoplasmic blush interpreted as a mutated pattern (cytoplasmic), or nuclear blush considered as a wild-type pattern instead of a mutated one (null). Training and awareness of different mutated patterns and staining artifact issues may reduce the misinterpretation rate of p53 staining [30,34]. 

MMR IHC displays three different patterns: retained, loss, or subclonal loss. While the retained pattern is the expression of a wild-type gene, loss may be due to somatic or germline mutation, and in the case of MLH1, also to promoter hypermethylation, and subclonal loss is commonly due to *MLH1* promoter mutation and expression of intratumoral somatic heterogeneity [32]. Interobserver reproducibility is very high for MMR IHC, and the reported discordance in interpretation is mainly due to intratumoral lymphocytes, heterogenous staining, and weak staining with reduced or absent positivity in the internal control [35].

To avoid misinterpretation, it is generally good practice to optimize the staining protocol, using appropriate negative and positive external controls; interpret staining expression with comparison to a positive internal control, such as stromal cells and lymphocytes; consider subclonal pattern, particularly in the case of morphologically heterogenous ECs; repeat IHC in a different paraffin block, especially from pre-operatory biopsy, or perform additional molecular workup in the case of equivocal or uninterpretable results [30,34].

Another relevant issue concerns the appropriate terminology to use in the pathologic report to avoid misunderstandings and confusion. P53 staining should be reported as the pattern type of staining, either wild-type or mutated/aberrant/abnormal, possibly followed by the specific type (overexpression, null, cytoplasmic, or subclonal), rather than meaningless negative or positive [34]. MMR IHC should be reported as either normal, complete loss, or subclonal/partial loss expression with the lost proteins specified and the conclusion mentioning the meaning in terms of clinical correlation [36,37]. The subclonal patterns of either p53 or MMR are strongly linked to MMRd and pathogenic *POLE* mutation; therefore, these possibilities, when unexplored, should be investigated [28,30].

#### 4.2.2. Diffusion and Accessibility

Nowadays, the portfolio of IHC in most conventional pathology laboratories includes hundreds of antibodies, comprising p53 and MMR proteins, which represent the most accessible tests for their determination [36]. Nevertheless, their utilization for EC molecular classification is not granted and is not even necessary in all cases (see Main Applications). A recent international survey of 69 European pathological laboratories distributed in Italy, Spain, Switzerland, and the UK showed that p53 IHC is performed in most laboratories: 67% in Switzerland, 83% in the UK, 80% in Italy, and 90% in Spain. Similarly, MMR IHC is available in 80% of Italian laboratories and in the totality of other participating laboratories [38]. However, it is performed in selected cases in 32% of Italian and 20% of Spanish laboratories, whereas it is performed in all cases in the other participating countries.

On the other side, the spread of *POLE* mutation testing remains limited, and *POLE* determination is not universally accessible because of the need for advanced infrastructure and trained personnel, so it is denied to most EC patients. Based on the previous survey, a minority of laboratories, 16 out of 69, performed *POLE* mutation analysis, including 10% of Italian, 50% of Spanish, and 67% of Swiss laboratories [38]. The survey covers a period between January 2020 and March 2021 and most likely underestimates the current situation, in which a larger number of laboratories may have adopted this test. A possible solution will be the centralization of POLE mutation analysis to selected reference laboratories, or the development of a valuable IHC surrogate.

#### 4.2.3. Non-Endometrioid Histotypes

The current WHO classification embeds molecular subtypes in endometrioid EC, the great majority of ECs, and suggests applying it in all ECs. Nevertheless, the clinical application of molecular subtypes indiscriminately to all histotypes seems at least premature. Among these, only serous EC was well represented in the TCGA study and was shown to be invariably molecularly characterized by a copy-number high. As a consequence, molecular characterization of serous EC could support pathological diagnosis, but given the molecular homogeneity, it seems irrelevant for further prognostication of this aggressive disease that is still grounded in traditional pathological features, such as degree of invasion, lymphovascular invasion, and tumor extension.

The prognostic and predictive impact of molecular subtyping for infrequent minor histotypes remains controversial, since few solid studies have been performed so far [39,40,41,42,43]. Indeed, the prognostic significance of TCGA molecular classification for different aggressive EC types, such as carcinosarcoma, clear cell, undifferentiated mixed cell, and neuroendocrine carcinoma, is generally supported. Specifically, clear cell EC is molecularly heterogeneous and is distributed across all four molecular subtypes [41,44]. Based on a limited number of cases and a univariate analysis, clear cell ECs with POLEmut and MMRd were associated with excellent outcomes as compared with p53mut and p53wt [41]. Two more recent studies did not observe significant survival differences in clear cell EC patients based on molecular subgroups [44,45]. On the other hand, Reijnen et al. found significant differences in OS and disease specific survival (DSS) of molecular subgroups in a cohort of patients with either clear cell EC or mixed EC with a clear cell component on both univariable and multivariable analyses [46]. Unfortunately, in the latter study, the histological heterogeneity of the population limits the validity of the conclusion.

Carcinosarcoma is another minor EC histotype, accounting for approximately 5% of all ECs; it molecularly mainly falls into the 53mut and NSMP groups and only sporadically falls into the POLEmut or MMRd molecular subtypes [40,47,48]. However, a very recent study found that all carcinosarcomas were exclusively p53mut, because the entire handful of molecularly non-p53mut were reclassified as pattern variants of endometrioid EC after the pathology review [49]. Once again, these findings highlight the importance of the correct pathological classification as the essential prerequisite of any further specification for prognostic and therapeutic assistance. Clinically, among the three original studies that investigated the impact of the molecular subtypes on carcinosarcoma patient prognosis, only one found significant improvement in both PFS and OS in MMRd patients compared with both the p53mut and NSMP subgroups and described an outstanding prognosis in POLEmut patients [39,47,50]. Moreover, a systematic review by Travaglino et al. similarly found excellent prognosis in POLEmut carcinosacoma patients and significantly improved PFS in MMRd carcinosarcomas by univariate analysis when compared with both p53mut and NSMP, but no OS [40]. However, the nature of the last study, pooling patient data from different studies, together with the limited number of patients in each group other than p53mut and the numerically unbalanced groups, seriously limits the consistency of these results.

Undifferentiated and dedifferentiated ECs, respectively, are completely or partially composed of an undifferentiated component of discohesive, monotonous, small- to intermediate-sized cells growing in patternless sheets [5,51]. They are aggressive tumors, accounting for up to 9% of ECs, but are generally underrecognized and misdiagnosed [52]. Molecularly, one-third to a half of these histotypes belong to the MMRd subgroup, followed by NSMP, with rare cases described as p53mut and POLEmut [43,53,54]. Among two studies that investigated the role of molecular group in the prognosis of undifferentiated or dedifferentiated histotypes, only one found that POLEmut ECs were significantly associated with improved DSS but no OS by univariable analysis [43,55]. The other study could not find significant differences in PFS and OS among the four molecular subgroups [55].

Mixed cell ECs seem to be distributed in all four molecular subgroups, with a prevalence of p53mut [6,56,57]. Several mixed ECs demonstrate a serous-like molecular profile identical in the different components, proving themselves misdiagnosed serous ECs with heterogenous or ambiguous morphology [56,57]. Nevertheless, a minority of cases are true mixed EC and show molecularly distinct features in the morphologically different components, due to either divergent or independent clonality. Usually, but not exclusively, they belong to either POLEmut or MMRd subgroups [28,30]. A study focused on women younger than 60 with EC with a serous phenotype, at least focal, found after histopathological review that several were mixed ECs with endometrioid and serous components [58]. The majority of these were MMRd or POLEmut and had a significantly better OS compared to the others, including bona fide serous ECs, but were not significant when compared only to other mixed ECs. Notably, in many cases, *POLE* mutation analysis was not performed.

The other infrequent histotypes, such as mesonephric-like, mesonephric, and mucinous ECs, should belong to NSMP or less frequently p53mut subgroups, while squamous cell EC usually harbors *TP53* and *CDKN2A* co-mutations making it p53mut, but evidence is still limited [59,60,61]. Finally, a recent study has detailed the molecular features of high-grade neuroendocrine ECs and classified them in all four molecular subgroups [42]. All these rare histotypes are considered aggressive, and the effect of molecular subgroups on clinical behavior has not been investigated yet.

In summary, to date, no convincing scientific evidence demonstrating the prognostic effect of molecular subgroups in special histotypes has been published; nevertheless, the POLEmut subgroup seems to bring a favorable outcome independently from histological features and histotype. Further supporting prospective investigations are still warranted to delineate this issue.

#### 4.2.4. NSMP

Importantly, the largest proportion of ECs are molecularly classified as NSMP (38–60%) and display an intermediate prognosis, including a wide spectrum of outcomes [62]. In high-risk endometrioid ECs, the NSMP subtype showed no significant differences in clinical progression compared to the p53mut subtype [23]. There is a cogent need to improve the prognostic stratification among NSMP patients.

Given that NSMP ECs are characterized by PIK3-AKT and WNT pathway activation, together with hormone receptor positivity, several studies have explored the utility of related, but also independent, molecular biomarkers as adjunct prognosticators to implement the molecular classification and differentiate NSMP patients with distinctive prognosis [6,63]. Specifically, based on previous studies, *CTNNB1*, *ARID1A* (or their IHC surrogates, b-catenin and ARID1A, respectively) mutations, *RAD51B* mutations, chromosome 1q32.1 amplification, FGFR2c expression, L1 cell adhesion molecule (L1CAM) IHC positivity, and progesterone receptor negativity could intercept NSMP endometrioid ECs with a worse prognosis [62,64,65,66,67,68,69,70]. Other studies identified a combination of molecular alterations as effective for prognostic stratification purposes in this molecular subtype [63,71,72].

In NSMP ECs, the combination of estrogen receptor (ER) status and histological grade allowed for the identification of very-low risk (low-grade ER-positive) and high-risk (grade 3 and/or ER-negative) subsets [73]. Moreover, in high-risk ECs, the only ER behaved as an independent prognostic factor in the NSMP subtype but not in the overall population [74]. In addition, ER status was an independent prognostic factor, also among patients with specific molecular ECs, in particular NSMP, MMRd, and p53mut; ER positivity (>10%) was associated with improved DFS and OS in all three subgroups [75].

#### 4.2.5. MMRd

The second most frequent molecular subtype is MMRd, which accounts for 20–40% of ECs and is associated with an intermediate prognosis analogous to NSMP [6]. Similarly, MMRd ECs generally have an endometrioid histotype but diversely carry worse pathological factors, such as high-grade, substantial lymphovascular space invasion, and higher FIGO stages [6,76,77]. They include hereditary syndromic cases linked to Lynch syndrome due to a germline mutation in one of the MMR genes [78]. In a few former studies, FGFR2c and L1CAM expression and ER negativity have been significantly associated with a poorer outcome among MMRd EC patients [68,70,75]. Further prognostic biomarkers are needed to allow for additional stratification and help clinical practitioners make care decisions for MMRd EC patients.

#### 4.2.6. The Prognostic Extremes: p53mut and POLEmut

The p53mut molecular group prognostically depicts the worst outcome among ECs, so that it manifests extraordinary dissemination risk even in the non-invasive phase [5]. Nevertheless, scientific evidence supports the contribution of clinico-pathological factors to the eventual outcome. In particular, traditional adverse clinico-pathological variables exacerbate the p53mut ominous prognosis; however, the role of the histotype remains contradictory [18,79,80,81].

The surprising novelty that has emerged from the TCGA study is the POLEmut group that behaves in an exceedingly favorable way, independently of both adjuvant therapy and several clinico-pathological prognostic factors, such as histologic grade, lymphovascular space invasion, and *TP53* mutation [80,82,83]. Notably, POLEmut ECs exhibit more frequently high-grade but limited myometrial infiltration, early stages (I–II), and maybe N0 [84,85,86]. Due to the limited numerosity of this subgroup and current available data, unanswered questions remain. These include whether POLEmut guarantees favorable prognosis also in non-endometrioid histotype, whether conservative surgery alone is a safe therapeutic option for all POLEmut patients or should be narrowed to a minority, and which ones, and which adjuvant therapy is more beneficial in POLEmut patients, among others.

### 4.3. Main Applications

The molecular classification demonstrates a valid diagnostic tool to define morphologically ambiguous and mixed cell ECs and to recognize endometrioid ECs with difficult patterns erroneously considered as other aggressive histotypes. As mentioned above, both serous EC and carcinosarcoma are almost invariably p53mut; therefore, in the case of alternative molecular results in these EC histotypes, a reconsideration of the histopathologic diagnosis is recommended.

Molecular classification is extremely useful in grade 3 endometrioid carcinomas, intercepting *POLE*-mut and p53-mut neoplasms, which represent the prognostic extremes of the molecular spectrum, with sometimes overlapping morphology but opposite implications in medical management [87,88]. Conversely, it is rather unnecessary in conventional serous EC or carcinosarcoma and low-stage, low-grade endometrioid EC that invariably belong to high-risk and low-risk prognostic groups, respectively.

Moreover, the application of universal MMR testing, recommended by most professional associations, allows for the identification of patients at risk for familial cancers and specifically affected by Lynch syndrome [78].

Finally, the most promising application of EC molecular classification concerns its ability to identify EC patients sensitive to and suitable for specific targeted therapies. Currently, this potentiality is largely explored in several ongoing clinical trials (see Ongoing and Future Perspectives). 

## 5. Standard Treatment

**Figure 2:**
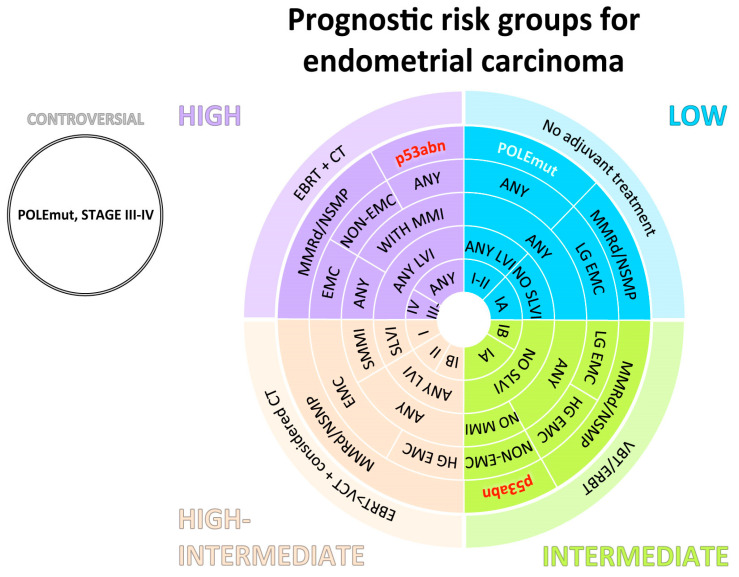
The risk groups for endometrial carcinoma without residual disease and therapeutic indications are shown as a concentrical graph [89]. Carcinosarcomas should be treated as high-risk carcinomas, not as sarcomas. p53abn EC without myometrial invasion belongs to the high-intermediate risk; for this risk group, further subclassification includes the pN0 stage versus lymph node staging not performed. For p53abn carcinomas restricted to a polyp or without myometrial invasion, adjuvant therapy is generally not recommended. Adjuvant chemotherapy can be considered, especially for HG and/or SLVI ECs. For patients with POLEmut EC, stage III–IVA, there are no outcome data with the omission of the adjuvant treatment. Prospective registration is recommended. Acronyms: VBT, vaginal brachytherapy; ERBT, external beam radiation therapy; CT, chemotherapy; MMRd, mismatch repair deficient; NSMP, no specific molecular profile; POLEmut, POLE-mutant; p53abn, p53 abnormal; EMC, endometrioid carcinoma. LG, low-grade; HG, high-grade; LVI, lymphovascular space invasion; SLVI, substantial LVI; MMI, myometrial invasion; SMMI, substantial MMI.

Current therapy guidelines include a combination of surgical and radiotherapy versus chemotherapy and/or radiotherapy based on the clinical, pathological, and molecular characteristics of the EC. Recently, the molecular classification was integrated into the prognostic risk stratification as a consensus by the European Society of Gynaecological Oncology (ESGO), the European Society for Radiotherapy and Oncology (ESTRO), and the European Society of Pathology (ESP), as a model to predict recurrence and possibly response to clinical therapy (Figure 2) [89]. Moreover, new target drugs are available according to the molecular subgroup classification.

An accurate family and personal medical history has to be collected in order to verify the possibility of Lynch syndrome [78].

### 5.1. Surgical Therapy

Total hysterectomy with bilateral salpingo-oophorectomy, often performed using a minimally invasive procedure such as laparoscopy or robotic assisted laparoscopy, is the treatment of choice for patients with EC. It might be complemented by lymphonodectomy depending on tumor characteristics (histotype, grade, stage, lymphovascular invasion), patients’ medical conditions (age, comorbidities), and national and international guidelines. Staging infracolic omentectomy should be performed in carcinosarcoma, serous, or undifferentiated EC, as well as peritoneal random biopsies.

Sentinel lymph node biopsy can be considered for staging purposes in patients with low-risk or intermediate-risk disease (stages I/II) [89].

### 5.2. Medical and Radiation Therapy

#### 5.2.1. Adjuvant Setting

Adjuvant therapy (AT) for EC is determined based on both stage and risk factors. While the FIGO staging system has traditionally guided treatment decisions, a more recent approach incorporates molecular characteristics to better classify EC risk and guide AT decisions. Radiation therapy, including pelvic external beam radiation therapy (EBRT) and vaginal brachiterapy (VBT), plays a crucial role in AT, particularly in lower-risk cases where most recurrences occur in the vaginal cuff. According to the latest NCCN guidelines, no AT is recommended for stage Ia G1-2 EC. For stage III and IV EC, systemic therapy with or without radiation therapy is recommended, while the addition of systemic therapy to EBRT or VBT may be discussed in selected cases. The recent ESGO/ESTRO/ESP prognostic risk stratification offers further guidance on AT; for example, suggesting the avoidance of chemotherapy in EC with a more favorable prognosis (Figure 2) [89]. In line with recent trials that highlighted the importance of molecular characteristics in guiding treatment decisions. In PORTEC-3, for example, comparing chemoradiation (CTRT) versus radiation (RT) alone in high-risk EC, the 5-year RFS rates differed based on molecular subtype, resulting in a significantly improved RSF in the adjuvant CTRT arm for p53abn EC but not in the POLEmut EC ones, which had a very favorable prognosis in both arms (17). Ongoing trials like PORTEC-4a have incorporated molecular EC characteristics to assess the safety and cost-effectiveness of AT in cases of favorable molecular profiles [90]. Platinum-based chemotherapy with or without paclitaxel remains the standard of care in cases where CTRT or systemic therapy is indicated for EC. These advancements in molecular profiling are enhancing personalized treatment approaches and improving outcomes for patients with EC.

#### 5.2.2. Recurrent and Metastatic Settings

Hormone therapy (HT) is often preferred as a front-line systemic therapy for low-grade and slow-growing ECs due to its scarce toxicity. Among different drugs, progestogens are usually the first choice, with aromatase inhibitors, tamoxifen, and fulvestrant being alternative options. According to a recent review, tamoxifen, either alone or in combination with progestogens, has higher response rates than aromatase inhibitors [91]. The general response rate to HT is reported to range from 15% to up to 55% in low-grade, endometrioid subtype, and hormonally dependent EC [89,92].

For recurrent as well as advanced EC, a platinum-based combination is the standard of care, as is surgery in selected cases. In patients with locoregional recurrence and no prior radiotherapy, the preferred primary therapy should be radiotherapy with or without chemotherapy. Despite the reported objective response rates (ORRs) of up to about 60% after first-line therapy, the duration of response and survival outcomes are poor [89,93,94]. For these reasons, many efforts are conducted, aimed at identifying new, effective drugs. Immune checkpoint inhibitors (ICIs) have emerged as promising options, particularly for patients with MSI-H or MMRd EC. Since the FDA approval of pembrolizumab in 2017, ICIs have shown safety and efficacy in recurrent or advanced EC, with ORR rates of up to about 60% and complete response rates of up to 16.3%, the highest across all tumor types [95]. Combination therapy involving ICIs and other drugs has also shown promise. In the KEYNOTE-775 trial, pembrolizumab and TKI lenvatinib produced a statistically significant OS advantage compared to chemotherapy (docetaxel or paclitaxel), regardless of MMR/MSI status.

To date, FDA-approved ICIs for previously treated MSI-H/MMRd EC include pembrolizumab and dostarlimab. Pembrolizumab in combination with lenvatinib is approved for mismatch repair-proficient/microsatellite-stable cases [96,97].

Bevacizumab, an anti-VGFR monoclonal antibody, has been studied in advanced EC, but with less favorable results. It is not licensed by the FDA for EC, and its use is only off-label, based on phase 2 study results. These studies showed a response rate of 13.5% and a 6-month PFS rate of 40.4%. Further studies and post hoc analysis have suggested a more favorable outcome for PFS and OS in the p53 mutant versus the p53wt ECs. This suggests that *TP53* mutation status may serve as a biomarker for ECs more likely to respond to bevacizumab treatment [95,98].

Multiple lines of therapy may be used in the treatment of metastatic EC, and a multidisciplinary professional team should be in charge of this care. Pembrolizumab-based anti-PD1 immunotherapy may be taken into consideration as a second-line treatment for MSI/MMRd carcinomas. Pembrolizumab with lenvatinib, a multi-tyrosine kinase inhibitor, may be used as a second-line therapy for microsatellite-stable carcinomas. Its usage, however, can be restricted because of national reimbursement laws or governmental authorization.

HER2, a receptor tyrosine-protein kinase encoded by *ERBB2*, is a well-known therapeutic target for many solid cancers, and potentially for EC too. IHC reports show high HER2/neu expression in about 35% of patients with uterine serous carcinoma, but to date, there is not a standardized scoring system for HER2 expression in EC [99,100,101].

According to a phase 2 trial by Fader et al., women with advanced or recurrent HER2 positive serous EC had significantly better OS (29.6 months vs. 24.4 months) and PFS (12.6 months vs. 8.0 months) when trastuzumab was added to carboplatin and paclitaxel chemotherapy [102]. Patients with stage III/IV illness showed the most benefit, indicating that HER2-targeted therapy would be a good course of treatment for some EC patient subgroups.

All patients with recurrence illnesses should be provided with the opportunity to participate in ongoing clinical trials.

## 6. Ongoing and Future Perspectives

### 6.1. Immunotherapy

A very interesting field of research is represented by ICIs in earlier stages of disease (adjuvant, neoadjuvant), as well as in fertility-sparing treatment. If in the metastatic setting the role of ICIs is well validated, in the earlier setting some concerns have been raised about a possible lower efficacy. Some data have revealed differences in the TME between earlier and advanced stage EC. In particular, at the early stage, ECs seem to show both lower PD-L1 expression, and intratumoral PD-1-positive/CD8-positive TILs, which could influence the response to such a therapy.

In the advanced or recurrent setting, identifying specific biomarkers with predictive value for treatment response is a significant challenge. Findings from the MITO-3 trial indicate that the *TP53* mutation is associated with a poor response to ICI avelumab, whereas mutations of *PTEN* and *ARID1A* are linked to a favorable response to this drug in patients with advanced EC [103]. These results underscore the importance of appropriately selecting patients for treatment, with significant implications for pharmacoeconomics and the avoidance of unnecessary toxicities.

### 6.2. PARP Inhibitors

The rationale for using PARP inhibitors (PARPi) in the treatment of EC stems from various pieces of evidence. Firstly, there are significant genomic similarities between p53abn EC and high-grade serous ovarian cancer, suggesting potential commonalities in their molecular pathways. Additionally, there is a potential impairment in homologous DNA recombination pathways, as indicated by the relatively high frequency of gene mutations, particularly in *TP53* and *PTEN*. Preliminary data have suggested a potential synergistic effect of PARPi when combined with ICIs. Preclinical models have shown increased PD-L1 expression and neoantigen load in response to PARPi treatment. However, the efficacy of this combination therapy has varied, with some studies reporting modest effects. The challenge in this complex molecular landscape lies in identifying a primary target to guide therapeutic decisions. Factors such as neoantigen load, PD-L1 expression, gene mutations, and impairment of DNA damage repair systems all contribute to the intricacy of the decision-making process. Further research is needed to clarify the most effective treatment strategies in this context [95]. Many ongoing studies have been designed to better assess the efficacy of PARPi with or without ICIs or other drugs in various disease settings, including maintenance therapy, recurrent disease, and different genetic backgrounds such as BRCA mutation carriers or BRCAwt.

### 6.3. Antibody–Drug Conjugates

Antibody–drug conjugates (ADCs) represent a significant advancement in the treatment of certain solid cancers. They consist of a highly selective monoclonal antibody directed against a specific tumor-associated antigen, linked to a cytotoxic drug via a chemical linker. The goal of ADC technology is to achieve potent tumor-killing activity while minimizing off-target effects. Several concluded and ongoing trials have explored ADCs targeting HER2 in various solid tumors, including breast cancer, uterine carcinosarcoma, and EC. The STATICE trial evaluates the efficacy of trastuzumab-deruxtecan (T-Dxd) in HER2-positive uterine carcinosarcoma, while the DESTINY-PanTumor02 trial enrolls patients with HER2-positive solid tumors [104]. Additionally, the ongoing NCT04585958 trial tests the combination of olaparib and T-DXd in HER2-expressing cancers, with expansion in patients with EC [105].

Although no ADC has been licensed to date, on December 2023, the FDA granted Breakthrough Therapy designation for BNT323/DB-130 (BioNTech, Mainz, Germany and DualityBio, Shanghai, China), an anti-HER2 antibody linked to a proprietary DNA topoisomerase I inhibitor, for the treatment of advanced HER-2 overexpressed EC in patients who progressed on or after treatment with ICI (https://prn.to/3R1NkIc, accessed on 28 June 2024). Further, on April 2024, the FDA granted accelerated approval to fam-trastuzumab deruxtecan-nxki (Enhertu, Daiichi Sankyo, Inc.) for adult patients with unresectable or metastatic HER2-positive (IHC 3+) solid tumors who have received prior systemic treatment and have no satisfactory alternative treatment options (https://www.fda.gov/drugs/resources-information-approved-drugs/fda-grants-accelerated-approval-fam-trastuzumab-deruxtecan-nxki-unresectable-or-metastatic-her2, accessed on 28 June 2024). In addition, ADCs targeting folate receptor alpha (FRα) have shown promise as potential therapeutic options for EC. FRα is overexpressed in approximately 64% of ECs, making it a potential target for ADC therapy, such as mirvetuximab and soravtansine [106]. Ongoing trials (such as NCT03835819 and NCT03748186) are investigating its efficacy, even if previous studies have shown modest results [107,108]. Another potential target for ADC therapy is the trophoblast cell surface antigen-2 (Trop2) frequently overexpressed in gynecological malignancies, in particular in over 90% of ECs [109,110]. One ADC-targeting Trop2 is sacituzumab-govitecan, which has been evaluated in a phase I/II basket trial assessing its efficacy and safety [111]. In the trial, it demonstrated an objective response rate (ORR) of 22.2% in EC. The median PFS was 3.2 months, and the median OS was 11.9 months.

### 6.4. ARID1A Inhibition

ARID1A belongs to the switch/sucrose non-fermenting (SWI/SNF) chromatin remodeling complex. *ARID1A* mutation is found in approximately 40% of ECs and causes a loss of function of ARID1A with a loss of protein expression [64]. As a consequence, the SWI/SNF complex is altered, contributing to cell cycle DNA checkpoint dysfunction and DNA damage, promoting migration and invasion [112,113].

Given its crucial functional role, targeting ARID1A may offer a promising approach to treating EC, particularly in patients harboring *ARID1A* mutations. This hypothesis is supported by preclinical and clinical data suggesting that ARID1A loss may confer sensitivity to certain therapeutic agents [112].

Tulmimetostat (CPI-0209) is a next-generation dual inhibitor of Enhancer of Zeste Homolog (EZH) 2 and EZH1. It is being investigated in a first-in-human, open-label sequential dose escalation and expansion phase 1/2 study (NCT04104776) in patients with advanced solid tumors and lymphomas, including *ARID1A*-mutated EC and ovarian clear cell carcinoma. Based on evidence from clinical trials, in September 2023, the FDA granted fast track designation for tulmimetostat for the treatment of patients with advanced, recurrent, or metastatic ARID1A mutated-EC and who have progressed on at least one prior line of treatment.

### 6.5. PI3K/AKT/mTOR Pathway Inhibitors

Dysregulation of the PI3K/AKT/mTOR pathway is frequently observed in most ECs and is associated with disease progression and resistance to anticancer drugs [114,115]. Drugs targeting this pathway represent a rational therapeutic option. In particular, for EC characterized by activation of the PI3K/AKT/mTOR pathway, usually endometrioid, these pathway inhibitors have been combined with hormone therapy (HT) with PI3K/AKT/mTOR pathway inhibitors or cyclin kinase inhibitors [116]. However, data from several studies investigating this combination therapy have been inconclusive. 

One phase II trial evaluating everolimus (a mTOR inhibitor) in combination with letrozole (EL) in hormone agnostic EC, showed a clinical benefit rate (CBR) of 40% and an objective response rate (ORR) of 32%, even in a cohort of only 35 patients [117].

In another study, the combination EL was compared to medroxyprogesterone acetate and tamoxifen (MT), revealing a similar response rate between the two groups (22% and 25%, respectively). However, a notable finding emerged regarding median PFS in EL-treated patients, which was significantly longer in chemotherapy-naive individuals compared to those who had received prior chemotherapy (28 months versus 4 months, respectively). This difference was not observed in the MT arm, where the median PFS was 5 months for chemotherapy-naive patients and 3 months for those with prior chemotherapy. This study also had a small cohort size of 37 patients in each treatment arm.

In a relatively recent review about PI3K/AKT/mTOR inhibitors by Roncolato et al., the authors concluded that only two randomized controlled trials have been reported, with low certainty of evidence [118]. Therefore, they conclude that a role of mTOR inhibitors could be observed in improving PFS in recurrent EC, but no reliable data are available regarding OS or tumor response rate.

New studies are ongoing, with many of them evaluating combination therapies involving aromatase inhibitors, everolimus, metformin, chemotherapy, and more.

A phase II, two-stage study testing letrozole/abemaciclib, a cyclin inhibitor, in recurrent ER-positive EC is ongoing, with ORR and PFS as primary endpoints [119]. An interesting aspect is a preliminary analysis of data at the cutoff date, which included exploratory tumor profiling. This analysis revealed several mechanistically possible candidate predictors of response (*CTNNB1*, *KRAS*, and *CDKN2A* mutations) or absence of response (*TP53* mutations), which require independent validation.

### 6.6. Other Drugs

The significant advancements in molecular and genetic research are introducing novel potential targets for future therapy options. Among ongoing studies, two recent ones stand out in the field of EC. The ENGOT-EN5/GOG-3055/SIENDO trial is a phase III study currently underway, evaluating the effects of Selinexor as a maintenance therapy following first-line chemotherapy for advanced/recurrent EC [120]. Selinexor is a first-in-class selective inhibitor of nuclear export, leading to the nuclear accumulation of tumor suppressor proteins, including p53. A preliminary analysis in a subgroup of patients with p53wt EC showed promising results with selinexor maintenance therapy.

The second study is a single-arm, two-stage phase II study that aims to investigate the effects of adavosertinib, a selective inhibitor of the WEE1 kinase, which is a key regulator of the G2/M and S phase cell-cycle checkpoints [121]. It has coprimary end points of ORR and six-month PFS, focusing on recurrent uterine serous carcinoma (USC). The rationale behind this approach is to target USC’s molecular characteristics, which involve cell-cycle dysregulation alongside a high level of oncogene-driven replication stress. Preliminary data suggest encouraging signs of durable adavosertinib activity.

### 6.7. Ongoing Trials Testing Tailored Therapies in Neoadjuvant/Adjuvant Setting

In confirmation of the importance of a better-integrated classification to provide the best-tailored therapy for women with not advanced EC, there are a significant number of ongoing clinical trials designed to approach this topic. A brief description of the most relevant trials, of which the majority have active recruiting, is displayed in Table 2.

## 7. Fertility Preservation

The growing research interest in molecular analysis and classification of EC, refining the group assignment, highlights the importance of reproductive considerations in these patients. Unfortunately, no strong evidence is currently available to guide decisions in this more recent context.

When EC is diagnosed in a fertile woman who expresses her conscious desire for pregnancy, organ preservation should be considered, though only in very selected settings. Among these are grade 1 neoplasms without myometrial invasion, no syndromic disease (e.g., Lynch syndrome, *BRCA* germline mutation carriers), p53wt, and L1CAM-negative cases [126].

Hormonal therapy with progestogens is the main treatment option for women eligible for fertility preservation. Since progesterone receptors are frequently highly expressed in very low-risk ECs, they usually respond well to such a therapy [127]. Different options include local and/or oral progestins, with or without gonadotropin-releasing hormone receptor agonists (GnRH-agonists).

The best recurrence-free rate is associated with hysterectomy, followed by progesterone therapy [128]. Moreover, these patients appear to have better OS and DFS when metformin is added to these medications [128]. A well-informed consensus, giving patient autonomy priority, and considering the specific oncological context, must be the prerequisite of any fertility consideration. Predictive factors for progestogen resistance are currently under study, and among these is MMRd status, one of the conditions effectively approached with ICIs [129,130,131,132].

Because NSMP tumors exhibit a variable prognosis, they require prognostic and predictive biomarkers for guiding therapy. Some ongoing trials aim to address this issue and clarify the implications of different molecular profiles on treatment strategies. These studies are critical in developing tailored therapeutic approaches, guiding fertility-sparing management, and optimizing outcomes for patients with different molecular subtypes of EC (Table 2) [133].

Although POLEmut ECs show a more favorable prognosis, the safety of conservative surgery alone for POLEmut EC is still to be verified, whereas p53abn tumors denote a poor prognosis, necessitating a strong reconsideration or avoidance of fertility-sparing options in such patients [90]. Coherently, ESGO guidelines indicate that conservative therapy is likely inappropriate [126].

## 8. Discussion

The detailed molecular characterization of cancer offers valuable improvements in many areas. Firstly, understanding the molecular landscape of cancer aids in comprehending both the natural history of the disease and the mechanisms involved in its development, chemosensitivity, and pharmacotherapy resistance. A better understanding of carcinogenesis, through the analysis of germline and somatic mutations, is not only relevant for the specific disease under investigation but also for all others that share the same molecular characteristics. Secondly, it allows for the identification of targetable molecular vulnerabilities and improves the prognosis and outcomes for patients thanks to the use of tailored therapies. Thirdly, it assists in preventing needless toxicities and expenses related to unsuccessful treatments.

The development of TCGA molecular classification for EC a decade ago and its rapid integration into clinical practice have given rise to a sudden explosion of studies and clinical trials to explore many aspects of its application, including methodology, integration with histological classification, prognostic and predictive value, implementation, and several others.

Many category associations recommend the application of molecular classification to all diagnosed ECs, but its effective utility in all histotypes remains controversial and not supported by solid scientific evidence. Certainly, molecular classification may integrate the histopathological diagnosis, confirming and refining histological classification, particularly for ECs morphologically mixed or ambiguous. However, its prognostic and predictive value for non-endometrioid EC needs to be consistently proven.

The ability of molecular classification to identify patients with POLEmut EC exhibiting excellent prognosis independently of other negative prognostic factors, including histologic grade, lymphovascular space invasion, *TP53* mutation, and likely histotype, remains of utmost importance. Although most pathology laboratories do not currently offer *POLE* mutation analysis since it requires expensive and advanced infrastructure; a reasonable solution will be to centralize *POLE* testing in selected landmark laboratories or develop novel, simpler surrogate tests. In addition, the real safety of chemotherapy de-escalation and conservative hysterectomy alone in POLEmut EC patients is still under consideration [90].

On the other hand, the wide range of outcomes within the most prevalent EC molecular subgroups, that is, NSMP and MMRd, urges the evaluation and incorporation of novel molecular biomarkers to better define the risk stratification and guide therapeutic strategies in these molecular subgroups. To this end, several ongoing clinical trials aim to test the molecular classification and each subgroup as a predictor of response to selected targeted therapies.

Importantly, traditional histopathological parameters remain the paramount prognostic factors in EC patients, confirming their prognostic significance also in all molecular subtypes, but in POLEmut in some instances [80]. Moreover, only careful and reliable morphological examination can identify prognostically significant features like lymphovascular space invasion, cervical and myometrial involvement, and unique growth patterns like microcystic, elongated and fragmented (MELF).

Finally, molecular classification does not offer additional aid for patients with advanced and recurring diseases, which remain one of the main clinical challenges in EC care.

## 9. Conclusions

In conclusion, despite the significant advancements in the EC field, from the Bokhman dualistic model to the latest integrated histo-molecular classification, many critical issues remain unmet. Among them is the prognostic impact of molecular subtype in non-endometrioid EC and its widespread application due to the unavailability of *POLE* mutation analysis in most territorial pathology services. The identification of specific biomarkers, both of drug resistance, especially for hormonal therapy, even in the maintenance setting, and of greater sensitivity to selected therapy options, which could allow for the safe avoidance of surgery in selected cases, is warranted. Similarly, more defined, molecular-driven guidelines are required for subsequent lines of therapy in advanced clinical settings. Not to be overlooked is the vital matter of fertility preservation.

The growing number of clinical trials in the adjuvant and metastatic settings of EC will likely produce evidence to refine most of these matters. Until then, the adjunct of molecular classification to conventional histological diagnosis is indispensable to arrive at an integrated histo-molecular classification of EC with demonstrated prognostic and predictive significance.

## Figures and Tables

**Table 1:** Evolution of the WHO classification of endometrial carcinoma.

1975 WHO Classification (1st Edition)	1994 WHO Classification (2nd Edition)	2003 WHO Classification (3rd Edition)	2014 WHO Classification (4th Edition)	2020 WHO Classification (5th Edition)
**Endometrial Carcinoma**				
Adenocarcinoma Clear cell (mesonephroid) adenocarcinoma Squamous cell carcinoma Adenosquamous [mucoepidermoid carcinoma] Undifferentiated carcinoma	Endometrioid adenocarcinoma Secretory (variant) Ciliated cell (variant) Adenocarcinoma with squamous differentiation (adenoacanthoma; adenosquamous carcinoma) Serous adenocarcinoma Clear cell adenocarcinoma Mucinous adenocarcinoma Squamous cell carcinoma Mixed carcinoma Undifferentiated carcinoma	Endometrioid adenocarcinoma Variant with squamous differentiation Villoglandular variant Secretory variant Ciliated cell variant Mucinous adenocarcinoma Serous adenocarcinoma Clear cell adenocarcinoma Mixed cell adenocarcinoma Squamous cell carcinoma Transitional cell carcinoma Small cell carcinoma Undifferentiated carcinoma Others	Endometrioid carcinoma Squamous differentiation Villoglandular Secretory Mucinous carcinoma Serous endometrial intraepithelial carcinoma (SEIC) Serous carcinoma Clear cell carcinoma Neuroendocrine tumors Low-grade neuroendocrine tumor Carcinoid tumor High-grade neuroendocrine carcinoma Small cell neuroendocrine carcinoma Large cell neuroendocrine carcinoma Mixed cell adenocarcinoma Undifferentiated carcinoma Dedifferentiated carcinoma	Endometrioid adenocarcinoma *POLE*-ultramutated endometrioid carcinoma Mismatch repair-deficient endometrioid carcinoma p53-mutant endometrioid carcinoma No specific molecular profile (NSMP) endometrioid carcinoma Serous carcinoma NOS Clear cell adenocarcinoma NOS Carcinoma, undifferentiated, NOS Mixed cell adenocarcinoma Mesonephric adenocarcinoma Squamous cell carcinoma NOS Mucinous carcinoma, intestinal type Mesonephric-like adenocarcinoma Carcinosarcoma NOS

Abbreviation: NOS, not otherwise specified.

**Table 2:** Ongoing clinical trials testing tailored therapies in neoadjuvant/adjuvant setting.

Trial	Acronym	Number	Type	Patients	Aim
Molecular Profile-based Versus Standard Recommendations for Adjuvant Radiotherapy for Women With Early Stage ECa [90]	PORTEC4a	NCT03469674	Prospective, multicenter, randomized phase III	High-intermediate risk EC	No adjuvant therapy, VBT or EBRT vs. standard adjuvant VBT
PErsonalized TReatment for EC [122]	PETREC	NCT05655260	Prospective, multicenter	Stage I–II high-intermediate or high-risk EC	Chemotherapy vs. chemoradiotherapy in p53abn and non-endometrioid ECs; VBT vs. EBRT in MMRd and NSMP
PROfiling-Based EC Adjuvant Therapy [123]	PROBEAT	NCT05179447	Prospective, multicenter, randomized phase III	High-intermediate and intermediate risk endometrioid ECs	No adj therapy, VBT, EBRT, or CTRT based on molecular features vs. standard RT
Refining Adjuvant Treatment IN ECa Based On Molecular Features [124] a. p53abn-RED trial b. MMRd EC to the MMRd-GREEN trial c. NSMP EC to NSMP-ORANGE trial d. POLEmut EC to the POLEmut-BLUE trial	RAINBO	NCT05255653	Umbrella program Randomized phase III Randomized phase III Randomized phase III Single arm phase II	Ecs eligible for adjuvant treatment	AdjCTRT + olaparib vs. adjCTRT adjEBRT + durvalumab vs. adjEBRT EBRT + Pg vs. CTRT Safety de-escalation
Tailored Adjuvant therapy in POLE-Mutated and p53-Wildtype/NSMP Early Stage EC a. EN10.A/RAINBO BLUE: POLE-mutated EC b. EN10.B/TAPER: p53 wildtype/NSMP EC		NCT05640999	Not randomized, open label phase III	POLE-mute or p53wt/NSMP (p53 wt/NSMP) EC	Testing de-escalated adj treatment
Letrozole as Maintenance Therapy for Post-surgical ECa Patients With NSMP		NCT05454358	Phase II/III open label, multicenter, superiority randomized controlled	NSMP surgically treated EC pts	Letrozole as maintenance therapy on the prognosis of post-operative NSMP EC
Neo-adjuvant Pembrolizumab as an Alternative Treatment for MMRd Uterine Cancer	PAM-II	NCT06180733	Phase II	Confirmed primary diagnosis of G3/CC MMRd EC who are intended to be treated with hysterectomy	To establish fraction of patients acquiring a MPR after nine cycles of pembrolizumab
Early Stage ECa Based on Molecular Classification and Traditional Risk Stratification to Guide Adjuvant Radiotherapy Decisions		NCT05524389	Phase III prospective, multicenter, randomized, open, non-inferiority	Stage I-II ECs	To assess 3-year LRR after adj RT based on molecular classification
Radiomics and Radiogenomics Models to Predict Molecular Integrated Risk Classes and Prognostic Factors in ECa	ROMANTIC	NCT06279832	Interventional	Stage IA/IB ECs	To develop radiogenomics models to stratify pts into three main risk categories according to the ProMisE model
Study and Transformation of Tumor Molecular Features Screening Model of EC Surgical Approach		NCT05894915	Prospective randomized controlled	ECs without high mutational burden characteristics (including *POLE* mutations, MSI-H, homologous recombinant repair pathway mutations)	To assess the impact of surgical routes on the short-term safety and long-term prognosis of EC pts with different molecular characteristics
Sentinel Lymph Node Sampling (SLN) for Patients With Middle-high Risk ECa Confined to the Uterus [125]	SNEC	NCT04276532	Non-inferiority randomized controlled	Intermediate-high risk EC	To investigate the effect of SLN sampling on the prognosis
Patient-derived Tumor-like Cell Clusters Predict Progesterone Sensitivity in Patients With Early ECa		NCT05647109	Observational	AEH/well-differentiated ECs G1 without myometrial invasion	To construct a prediction model of Pg sensitivity in pts with EC treated with fertility preservation
feasibility safety Efficacy dostarLimab earLy-stage defIcient endomeTrial cancEr	SATELLITE	NCT06278857	Phase IIb, open-label, single arm, multicenter, pilot study	Early-stage MMRd EC	Dostarlimab as a potential alternative to surgery

Abbreviation: VBT, vaginal brachytherapy; EBRT, external beam radiation therapy; CTRT, chemoradiotherapy; RT, radiotherapy; ECa, endometrial cancer; EC, endometrial carcinoma, adj: adjuvant; CC, clear cell; LRR, local recurrence rate; pts, patients; MPR, major pathological response; ProMisE, Proactive Molecular Risk Classifier for EC; AEH, atypical endometrial hyperplasia; MMRd, mismatch repair deficient; NSMP, no specific molecular profile; POLEmut, POLE-mutant; p53abn, p53 abnormal.
